# TBL alone or combined with other teaching methods for neurology education in China: a meta-analysis of randomized controlled trials

**DOI:** 10.1186/s12909-026-09468-1

**Published:** 2026-05-22

**Authors:** Jie Fu, Xiu Chen, Jinglun Li, Lilei Peng

**Affiliations:** 1 Department of Neurology, The Affiliated Hospital of Southwest Medical University, Taiping Street, Jiangyang District, Luzhou, 646000 Sichuan China; 2 Department of Neurosurgery, The Affiliated Hospital of Southwest Medical University, Taiping Street, Jiangyang District, Luzhou, 646000 Sichuan China

**Keywords:** Meta-analysis, Neurology, Team-based learning, Lecture-based learning

## Abstract

**Background:**

Team-based learning (TBL) has been applied in neurology teaching. This meta-analysis aimed to compare the effects of TBL alone or in combination with other methods versus lecture-based learning (LBL) on improving the teaching effects of neurology in China.

**Methods:**

A comprehensive literature search was performed to obtain randomized controlled trials (RCTs) assessing the efficacy of TBL and LBL on neurology education in China. The Review Manager 5.2 software and STATA 15.0 software were employed for this meta-analysis of the included studies.

**Results:**

A total of 16 studies involving 1267 medical students were included. The pooled results showed that compared to traditional LBL, TBL was related to higher theoretical knowledge scores and practical skill scores (SMD = 1.16, 95% CI: 0.77 to 1.54, *p* < 0.00001; SMD = 1.72, 95% CI: 1.19 to 2.24, *p* < 0.00001; respectively). Additionally, our results demonstrated the efficacy of TBL in enhancing students’ self-study ability, communication skills, and teamwork ability (SMD = 1.15, 95% CI: 0.69 to 1.61, *p* < 0.00001; SMD = 2.36, 95% CI: 0.74 to 3.98, *p* = 0.004; SMD = 0.97, 95% CI: 0.68 to 1.26, *p* < 0.00001; respectively ). Meanwhile, students in the TBL group were more satisfied than those in the LBL group.

**Conclusions:**

Our data suggest that TBL-based teaching approaches (including TBL alone and TBL combined with other methods) may be more effective than LBL in enhancing theoretical knowledge scores and practical skill scores in Chinese neurology education. Additionally, TBL may be associated with improved self-study capacity, communication skills, teamwork ability as well as student satisfaction. However, considering the limitations such as the low methodological quality of the included studies and obvious heterogeneity, future multicenter, cross-cultural and high-quality studies are needed to validate and generalize our findings.

**Supplementary Information:**

The online version contains supplementary material available at 10.1186/s12909-026-09468-1.

## Background

Neurology is a second-level clinical discipline that focuses on the diagnosis and treatment of neurological diseases, which is widely considered as one of the toughest medical specialties [1, 2]. Students are often bombarded with a large amount of learning material covering the complex anatomy of the nervous system and a broad spectrum of neurological disorders, which leads to their fear of neurology and neuroscience, a widely reported phenomenon named “neurophobia” [3]. Neurophobia may result in anxiety, aversion, and lack of interest in the subject [4], which has implications on the practice of neurology, finally influencing the quality of care for patients with neurological disorders [5]. Thus, addressing neurophobia and improving neurology education are essential, which contributes to better preparing students for subsequent clinical practice, further improving the quality and safety of medical care.

In China, the teaching of neurology still primarily uses traditional lecture-based learning (LBL) method. In this teaching mode, teachers deliver theoretical knowledge to students in a classroom setting within a limited time period. This traditional pedagogical style is problematic due to its constraints on student interaction and practical applications of theoretical knowledge, which consequently inhibits the stimulation of active learning and critical thinking skills as well as results in a theory-practice gap [6, 7]. However, neurology is a complex, rapidly evolving field that requires more than just memorization of neuroanatomy and disease facts, which demands clinical reasoning, problem-solving, and effective teamwork. Thus, new teaching approaches are needed to enhance the efficacy of neurology education. In recent years, researchers have performed various studies on the reform of teaching methods in neurology. Team-based learning (TBL) is a teacher-directed small-group learning strategy that engages students in both individual and group tasks [8], which was first introduced in 1970 [9]. TBL creates a dynamic learning environment, and promotes active student participation, analytical thinking as well as knowledge retention, which has been adopted by many medical schools [10]. Prior investigations have indicated that students in TBL groups achieve superior scores on theoretical knowledge and practical skills than those in traditional LBL groups [11, 12]. In addition to improving theoretical and practical performance, TBL is beneficial for developing students’ multiple abilities including self-study and teamwork, and enhancing students’ class engagement and satisfaction [13]. All in all, TBL moves education from the passive transmission of information to the active cultivation of clinical problem-solvers. By emphasizing application, teamwork, and communication, TBL prepares students not only to acquire the basic medical knowledge but to address clinical issues in a collaborative clinical environment. Thus, TBL may be more adaptable to the requirements of modern neurology education.

However, current studies related to TBL approach on neurology teaching are frequently limited by small sample sizes and single-center designs. Additionally, some studies have yielded inconsistent results. To address these issues, we performed a meta-analysis to compare the teaching efficacy of TBL or TBL combined with other teaching methods with traditional LBL on neurology education in China.

## Methods

Our meta-analysis was carried out according to the recommendations of the Preferred Reporting Items for Systematic Reviews and Meta-Analyses (PRISMA) [14] statement (Supplementary file 1). Ethical approval was not required because our study was retrieved based on previous literature data. The protocol for this meta-analysis was registered on the International Platform of Registered Systematic Review and Meta-analysis Protocols (INPLASY). The unique registration number is INPLASY202650038 (DOI: 10.37766/inplasy2026.5.0038).

### Search strategy

Pubmed, Cochrane Library, Web of Science, China National Knowledge Infrastructure (CNKI), Chinese VIP database and Chinese Wanfang Database were searched up to July 2025. The search terms used were as follows: (“team-based learning” OR “TBL” ) AND (“lecture-based learning” OR “LBL” OR “traditional teaching”) AND “neurology”. Furthermore, the search strategy was modified to comply with the requirements of different databases. The detailed search strategy was shown in Supplementary file 2. Prior reviews and meta-analyses were also searched to identify additional studies. The articles published in English or Chinese were included.

### Selection criteria

We defined the inclusion criteria based on the evidence-based PICOS principles and the criteria were outlined below: (1) P (participant): campus-based students receiving neurology education as well as rotating interns and residents in Neurology in mainland China; (2) I (intervention): TBL or TBL combined with other teaching methods; (3) C (comparison): traditional LBL approach; (4) O (outcome): theoretical knowledge scores or practical skill scores; (5) S (study design): randomized controlled trials (RCTs). The exclusion criteria were as follows: (1) duplicate publications; (2) lack of availability of full text or outcome data; (3) reviews, abstracts, meta-analyses and expert opinions. Two researchers (FJ and LJL) independently screened the studies for eligibility. A third researcher (PLL) was consulted to resolve any discrepancies.

### Data extraction

Two researchers (LJL and CX) independently extracted the following data from all included studies according to the selection criteria mentioned above: author, publication year, sample size, study design, age, gender, intervention methods, outcomes, and intervention duration. The primary outcome was theoretical knowledge scores or practical skill scores. Secondary outcomes included self-study capacity, communication skills, teamwork ability as well as student satisfaction.

### Quality assessment

The Cochrane Collaboration’s risk of bias tools were applied to evaluate the risk of bias of all included RCTs. The criteria included random sequence generation, concealment of allocation, blinding of participants and outcome assessment, incomplete outcome data, selective reporting, and other sources of bias [15]. The results of the assessment were classified as low, unclear, or high risk of bias.

### Statistical analysis

This meta-analysis was performed by using the Review Manager 5.2 software and STATA 15.0 software. The outcomes were expressed as standardized mean differences (SMD) for continuous variables and odds ratios (ORs) for dichotomous variables, with 95% confidence intervals (CI). The Chi2 tests and the *I*^2^ statistic were adopted to assess the heterogeneity among the contrasts. The studies were regarded significantly heterogeneous if *I*^2^ > 50%, and a random-effects model was applied. Otherwise, a fixed-effects model was used. We applied meta-regression analyses to explore the sources of high heterogeneity. Subgroup analyses were conducted to assess differences in effects based on intervention methods, intervention duration and types of participants. Sensitivity analyses were performed to evaluate the reliability of the results. Publication bias was assessed by visual inspection of a funnel plot, and further checked by Egger’s test and the trim-and-fill method. *p*-value < 0.05 in Z test was considered statistically significant.

## Results

### Literature search results and characteristics of the included studies

**Fig. 1:**
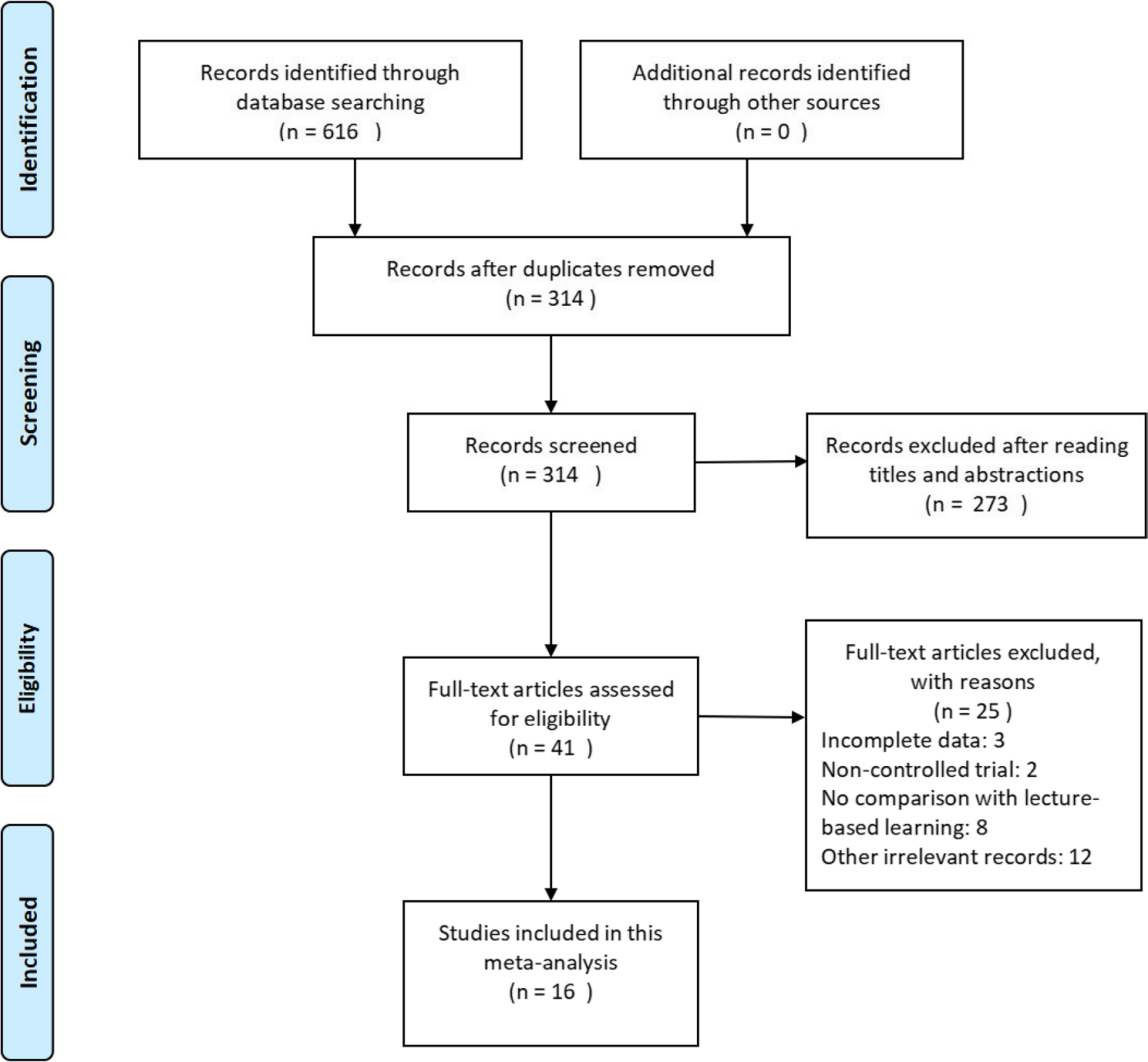
Flow chart of the literature screening and selection process

The search flow diagram was presented in Fig. 1. Six hundred and sixteen literature were identified from the initial search. 302 duplicates were ruled out and 41 full-text articles were selected for further appraisal. 16 studies with 1267 subjects (634 in the TBL or combined TBL group and 633 in LBL group) were ultimately included in the meta-analysis [16–31]. The sample sizes of included studies ranged from 30 to 200. The students’ theoretical knowledge scores were reported in 15 of the studies, the students’ practical skill scores were assessed in 14 studies, and self-study ability, communication skills, teamwork ability, and satisfaction were investigated in 3, 4, 3, and 10 studies, respectively. The intervention group in 9 studies only adopted TBL teaching method, and the intervention group in other 7 studies applied TBL combined with other teaching methods. The characteristics of the included studies are shown in Table 1.

**Table 1:** Characteristics of the included studies

Study	Sample size		Age (year)		Gender (M/F)		Participants	Interventions		Outcomes	Duration of intervention
	TBL group	Control group	TBL group	Control group	TBLgroup	Controlgroup		TBLgroup	Controlgroup		
Zhang et al [16]	100	100	23.76 ± 0.88	23.72 ± 0.89	56/44	53/47	Students on campus	TBL	LBL	①②③	Not mentioned
Lei et al [17]	47	46	21.3 ± 0.2	21.4 ± 0.1	25/22	24/22	Students on campus	TBL	LBL	①	Not mentioned
Wang et al [18]	15	15	—		—		Students on campus	TBL	LBL	①②③	Not mentioned
Rong et al [19]	40	40	20.79 ± 1.45	22.18 ± 1.52	—		Students on campus	TBL	LBL	①②	Not mentioned
Yin [20]	15	15	—		—		Residents	TBL	LBL	②	2 months
Yang et al [21]	43	43	22.0 ± 1.76	21.6 ± 2.25	21/22	23/20	Students on campus	TBL	LBL	①②	2 weeks
Qin et al [22]	50	50	23.34 ± 1.12	23.4 ± 0.88	30/20	28/22	Students on campus	TBL+Internet	LBL	①②③	1 year
Fan et al [23]	33	33	22.27 ± 0.81	22.47±0.76	14/19	15/18	Interns	TBL	LBL	①②③④⑤⑥	Not mentioned
Fan (2) et al [25]	27	27	22 .15 ± 0 .92	22.26 ± 0 .85	14/13	13/14	Interns	TBL	LBL	①②④⑤⑥	Not mentioned
Zhang et al [24]	36	36	22.3 ± 2.0	21.9 ± 2.1	18/18	17/19	Interns	TBL	LBL	①②③⑤	Not mentioned
Xu et al [26]	39	39	—		35/43		Interns	TBL+CP	LBL	①②③	4 weeks
Gao et al [27]	40	40	20~24	19~24	18/22	19/21	Interns	TBL+TNS	LBL	①②③④⑤⑥	Not mentioned
Zhao et al [28]	30	30	—		—		Students on campus	TBL+PBL	LBL	①②③	Not mentioned
Wang et al [29]	59	59	—		49/69		Students on campus	TBL+CBL	LBL	①	Not mentioned
Liu et al [30]	20	20	24.43 ± 2.33	23.78 ± 3.12	8/12	7/13	Residents	TBL+flipped classrooms	LBL	①②③	1 semester
Hou et al [31]	40	40	—		—		Students on campus	TBL+CBL	LBL	①②③	Not mentioned

① theoretical knowledge scores; ②practical skill scores; ③ student satisfaction; ④ self-study ability; ⑤communication skills; ⑥teamwork ability

*LBL* lecture-based learning, *TBL *team-based learning, *M *male, *F *female, *CP *clinical pathway, *TNS *Tutor-Network-Seminar, *CBL *case-based learning, *PBL *problem-based learning

### Quality assessment of the included literature

**Fig. 2:**
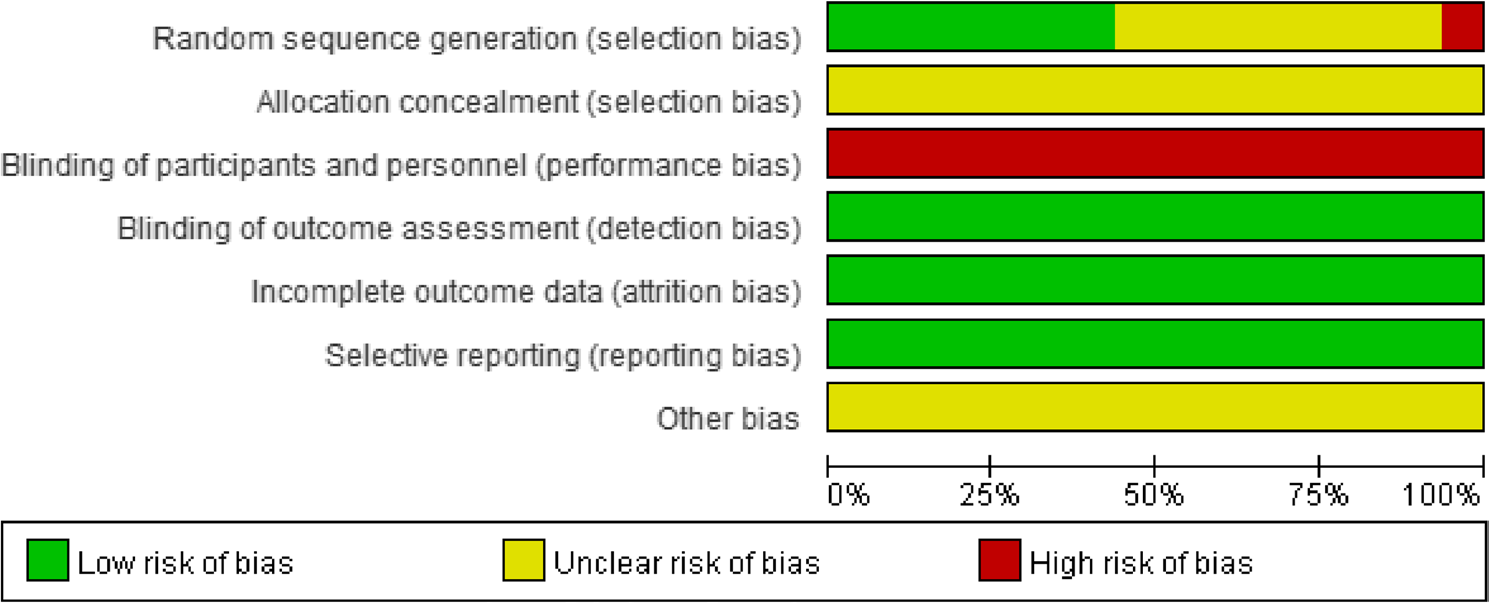
Risk of bias graph of all included studies

We employed the Cochrane risk-of-bias tool to assess the quality of all included studies. Among the 16 studies included, 7 studies adopted an appropriate random sequence generation method and were assessed as low risk. One study described the method as “randomized”, but participants were divided into intervention and control groups by even and odd numbers, and it was assessed as high risk, other 8 studies did not clearly mention the randomization process and were assessed as unclear risk. None of the studies indicated allocation concealment. Due to the characteristics of the teaching process, blinding of teachers and students was not feasible. All studies were judged to be at low risk of the outcome assessment. In addition, all studies reported all outcomes and were not selectively reported. Other sources of bias were unclear. The results of the risk of bias of included studies were displayed in Fig. 2.

### Meta-analysis results of theoretical knowledge scores

**Fig. 3:**
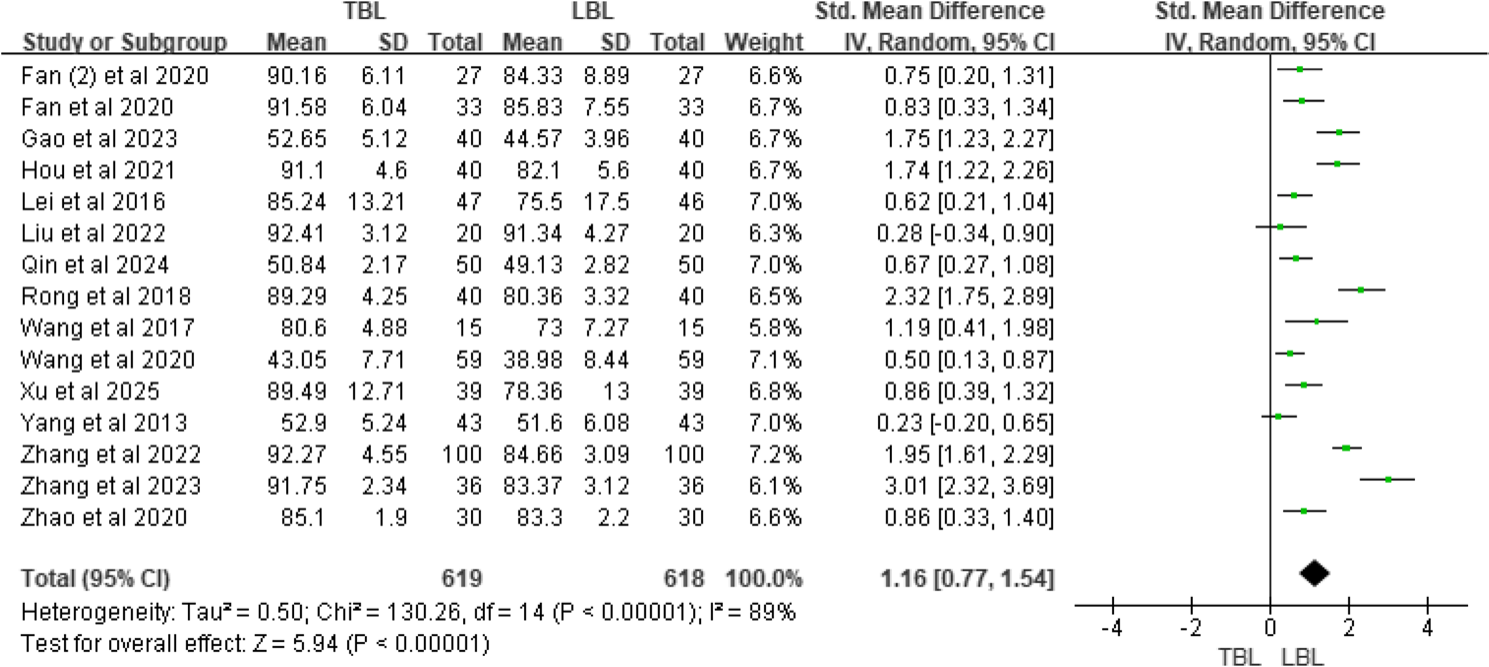
Forest plot of theoretical knowledge scores for TBL compared with LBL. CI = confidence interval, SD = standard deviation, LBL = lecture-based learning, TBL = team-based learning

This meta-analysis included 15 studies that investigated the theoretical knowledge scores of 1237 students, including 619 students in the intervention group and 618 students in the control group. The random effects model was employed for the meta-analysis due to obvious heterogeneity among the studies (*I*^2^ = 89%). The results indicated that compared to traditional LBL, TBL was related to higher theoretical knowledge scores (SMD = 1.16, 95% CI: 0.77 to 1.54, *p* < 0.00001) (Fig. 3).

Meta-regression analysis was employed to investigate the sources of heterogeneity among the included studies. Theoretical knowledge scores served as the outcome variable, while intervention methods, intervention duration and types of participants were used as predictive variables. The results demonstrated that none of the three variables contributed remarkably to the heterogeneity in theoretical knowledge scores (intervention methods: β = -0.26, *p* = 0.54; intervention duration: β = 0.49, *p* = 0.12; participants: β = 0.22, *p* = 0.61; Table 2).

**Table 2:** Results of meta-regression

Index	Variable	Coefficients	95%CI	*p*-value
Theoretical knowledge				
	Intervention methods	−0.26	−1.17 ~ 0.65	0.54
	Intervention duration	0.49	−0.15 ~ 1.12	0.12
	Participants	0.22	−0.70 ~ 1.13	0.61
Practical skills				
	Intervention methods	−0.62	−2.35 ~ 1.11	0.44
	Intervention duration	0.73	−0.44 ~ 1.90	0.20
	Participants	0.28	−1.41 ~ 1.98	0.72

Subgroup analyses were performed to examine whether the effects varied by study characteristics including intervention methods, intervention duration and types of participants (Supplementary Fig. 1). In the subgroup analysis stratified by intervention methods (TBL alone vs. TBL combined with other methods), both subgroups demonstrated significantly better outcomes for TBL group compared to traditional LBL group. The pooled effect size was SMD = 1.35 (95% CI: 0.71 to 1.99) for TBL alone subgroup and SMD = 0.95 (95% CI: 0.54 to 1.35) for blended TBL subgroup, and no significant difference was detected between the subgroups (*p* = 0.30). Additionally, obvious heterogeneity was found between the associated studies (*I*^2^ = 92%; *I*^2^ = 80%; respectively). In the subgroup analysis according to intervention duration (≤ 4 weeks vs. > 4 weeks vs. Not reported), ranked by effect size from largest to smallest, the results were: Not reported (SMD = 1.40, 95% CI: 0.94 to 1.85), > 4 weeks (SMD = 0.55, 95% CI: 0.19 to 0.91) and ≤ 4 weeks (SMD = 0.53, 95% CI: -0.08 to 1.15), and the subgroup difference was statistically significant (*p* = 0.01). Notably, in the > 4 weeks subgroup, the heterogeneity decreased substantially (*I*^2^ = 8%). In contrast, the ≤ 4 weeks subgroup and Not reported subgroup still exhibited considerable heterogeneity (*I*^2^ = 74%; *I*^2^ = 89%; respectively). When stratifying by types of participants (Students on campus vs. Interns or residents), both subgroups demonstrated significantly better outcomes for TBL group compared to traditional LBL group with high heterogeneity between the associated studies. The pooled effect size for the Students on campus subgroup was SMD = 1.11 (95% CI: 0.62 to 1.60) with considerable heterogeneity (*I*^2^ = 90%). Similarly, the Interns or residents subgroup showed an effect size of SMD = 1.23 (95% CI: 0.55 to 1.91) and also exhibited substantial heterogeneity (*I*^2^ = 89%). The test for subgroup difference was not significant (*p* = 0.78).

**Fig. 4:**
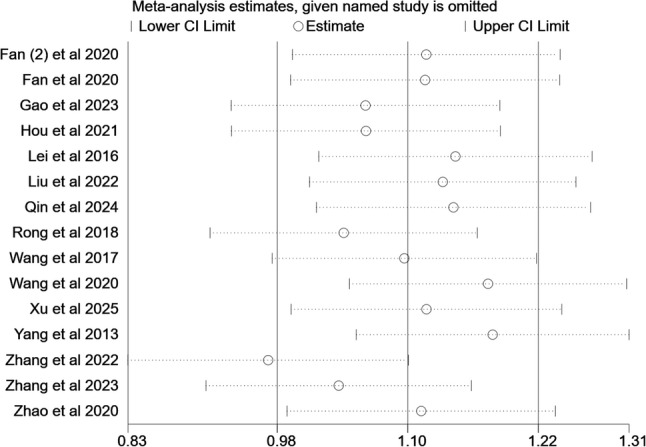
Sensitivity analysis of theoretical knowledge scores for TBL compared with LBL. CI = confidence interval, LBL = lecture-based learning, TBL = team-based learning

The sensitivity analysis revealed that the omission of any single study did not significantly change the combined SMD, suggesting that the observed effects were not solely attributable to any single included study (Fig. 4).

### Meta-analysis results of practical skill scores

**Fig. 5:**
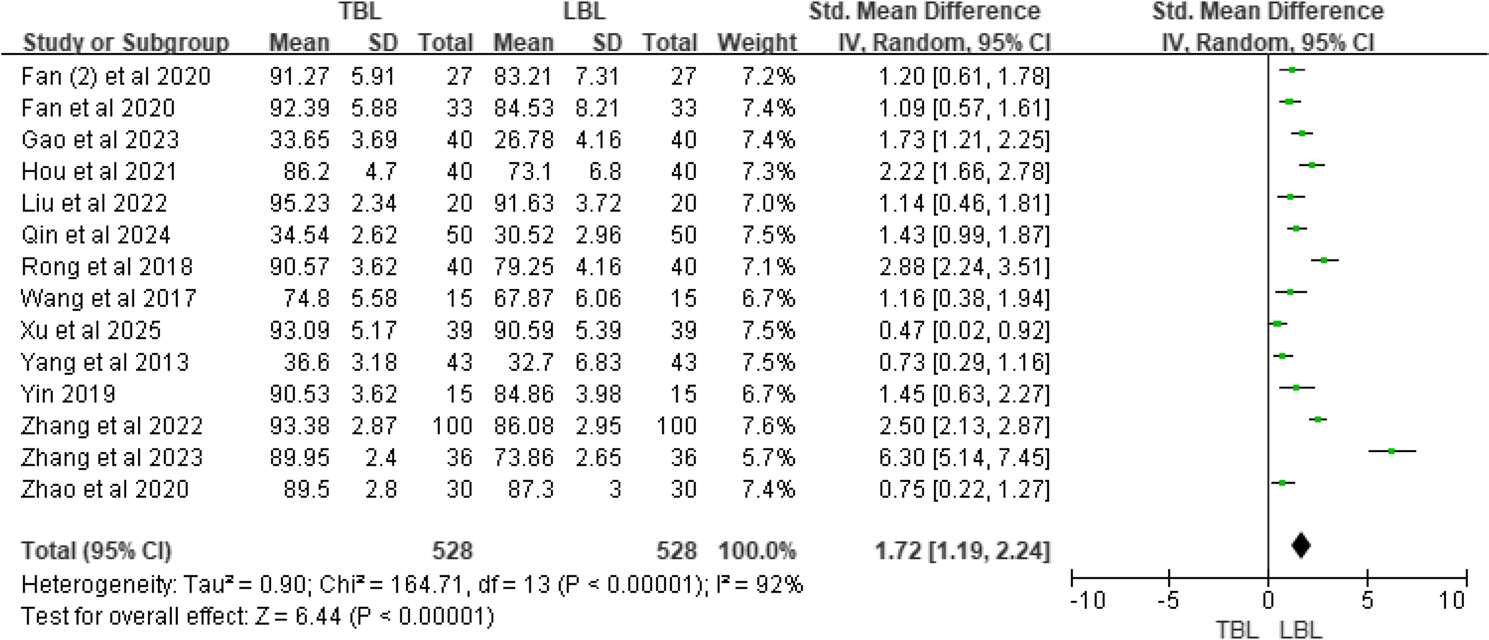
Forest plot of practical skill scores for TBL compared with LBL. CI = confidence interval, SD = standard deviation, LBL = lecture-based learning, TBL = team-based learning

This meta-analysis included 14 studies that investigated practical skill scores of 1056 students, including 528 students in the intervention group and 528 students in the control group. The random effects model was employed for the meta-analysis due to obvious heterogeneity among the studies (*I*^2^ = 92%). The results indicated that compared to traditional LBL, TBL was associated with higher practical skill scores (SMD = 1.72, 95% CI: 1.19 to 2.24, *p* < 0.00001) (Fig. 5).

Meta-regression analysis was employed to investigate the sources of high heterogeneity among the included studies. Practical skill scores served as the outcome variable, while intervention methods, intervention duration and types of participants were used as predictive variables. The results demonstrated that none of the three variables contributed remarkably to the heterogeneity in practical skill scores (intervention methods: β = -0.62, *p* = 0.44; intervention duration: β = 0.73, *p* = 0.20; participants: β = 0.28, *p* = 0.72; Table 2).

Subgroup analyses were performed to examine whether the effects varied by study characteristics including intervention methods, intervention duration and types of participants (Supplementary Fig. 2). In the subgroup analysis stratified by intervention methods (TBL alone vs. TBL combined with other methods), both subgroups demonstrated significantly better outcomes for TBL group compared to traditional LBL group. The pooled effect size was SMD = 2.09 (95% CI: 1.23 to 2.94) for TBL alone subgroup and SMD = 1.28 (95% CI: 0.76 to 1.81) for blended TBL subgroup, and no significant difference was detected between the subgroups (*p* = 0.12). Additionally, obvious heterogeneity was found between the associated studies (*I*^2^ = 94%; *I*^2^ = 84%; respectively). In the subgroup analysis according to intervention duration (≤ 4 weeks vs. > 4 weeks vs. Not reported), ranked by effect size from largest to smallest, the results were: Not reported (SMD = 2.12, 95% CI: 1.40 to 2.84), > 4 weeks (SMD = 1.36, 95% CI: 1.02 to 1.69) and ≤ 4 weeks (SMD = 0.60, 95% CI: 0.29 to 0.91), and the subgroup difference was statistically significant (*p* < 0.0001). Notably, the heterogeneity was fully resolved in both the ≤ 4 weeks subgroup and > 4 weeks subgroup (both *I*^2^ = 0%). In contrast, the Not reported subgroup still exhibited considerable heterogeneity (*I*^2^ = 93%). When stratifying by types of participants (Students on campus vs. Interns or residents), both subgroups demonstrated significantly better outcomes for TBL group compared to traditional LBL group with high heterogeneity between the associated studies. The pooled effect size for the Students on campus subgroup was SMD = 1.66 (95% CI: 1.01 to 2.32) with considerable heterogeneity (*I*^2^ = 91%). Similarly, the Interns or residents subgroup showed an effect size of SMD = 1.81 (95% CI: 0.92 to 2.70) and also exhibited substantial heterogeneity (*I*^2^ = 93%). The test for subgroup difference was not significant (*p* = 0.80).

**Fig. 6:**
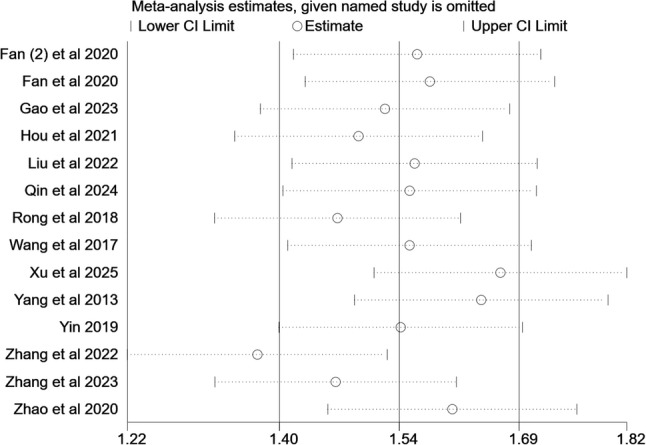
Sensitivity analysis of practical skill scores for TBL compared with LBL. CI = confidence interval, LBL = lecture-based learning, TBL = team-based learning

The sensitivity analysis by sequentially excluding each study showed that no single study substantially influenced the total SMD, indicating that the overall findings were not solely driven by any individual study (Fig. 6).

### Meta-analysis results of secondary outcomes

**Fig. 7:**
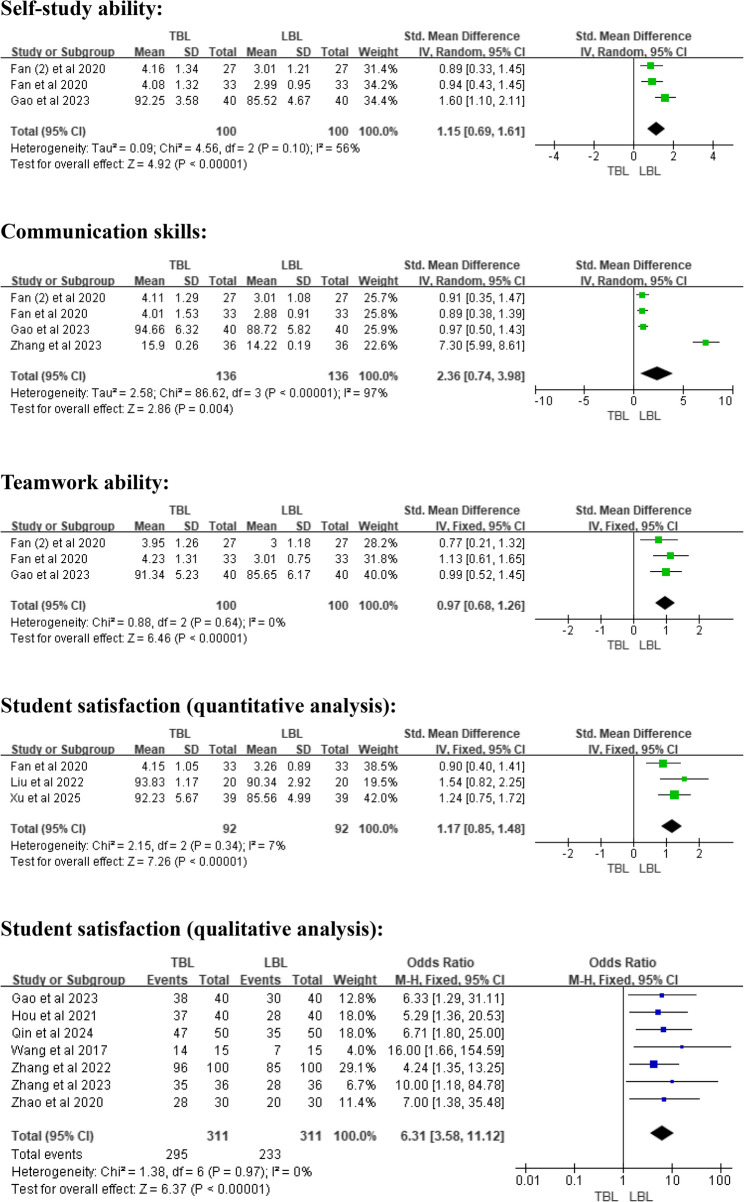
Forest plots of self-study ability, communication skills, teamwork ability, and student satisfaction for TBL compared with LBL. CI = confidence interval, SD = standard deviation, LBL = lecture-based learning, TBL = team-based learning

We further assessed the effects of TBL teaching method on secondary outcomes including self-study capacity, communication skills, teamwork ability, as well as student satisfaction (Fig. 7). For self-study ability, 3 studies were included in the meta-analysis, and the pooled SMD was 1.15 (95% CI: 0.69 to 1.61, *p* < 0.00001). For communication skills, 4 studies were included, and the meta-analysis yielded a SMD of 2.36 (95% CI: 0.74 to 3.98, *p* = 0.004). For teamwork ability, 3 studies were included in the meta-analysis, and the pooled SMD was 0.97 (95% CI: 0.68 to 1.26, *p* < 0.00001). These results indicated the efficacy of TBL in enhancing students’ self-study ability, communication skills, and teamwork ability.

For student satisfaction, 3 studies reported continuous data, while 7 studies reported dichotomous data, and thus we conducted the meta-analysis separately. The pooled SMD of satisfaction with continuous data was 1.17 (95% CI: 0.85 to 1.48, *p* < 0.00001), and the overall OR for satisfaction with dichotomous data was 6.31 (95% CI: 3.58 to 11.12, *p* < 0.00001). These results suggested that students in the TBL group were more satisfied than those in the LBL group.

### Publication bias

**Fig. 8:**
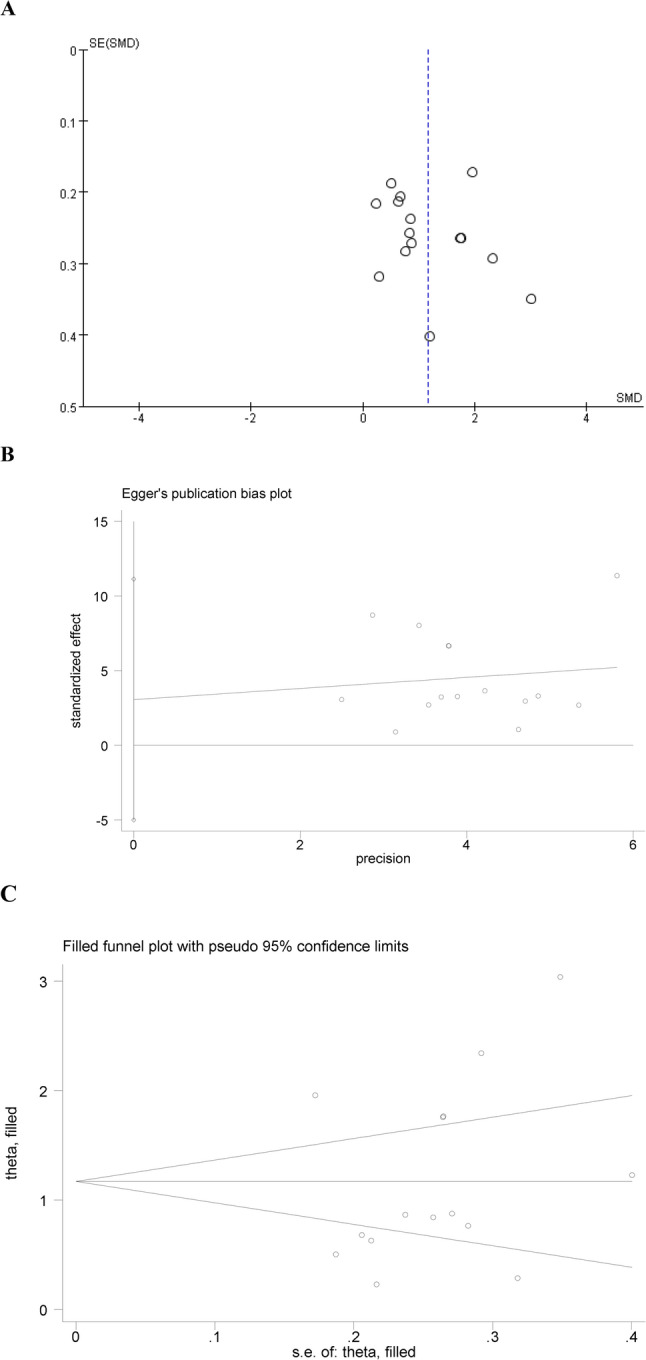
The funnel plot (**A**), Egger’s test (**B**), and trim and fill plot (**C**) of theoretical knowledge scores. SE = standard error, SMD = standardized mean difference

**Fig. 9:**
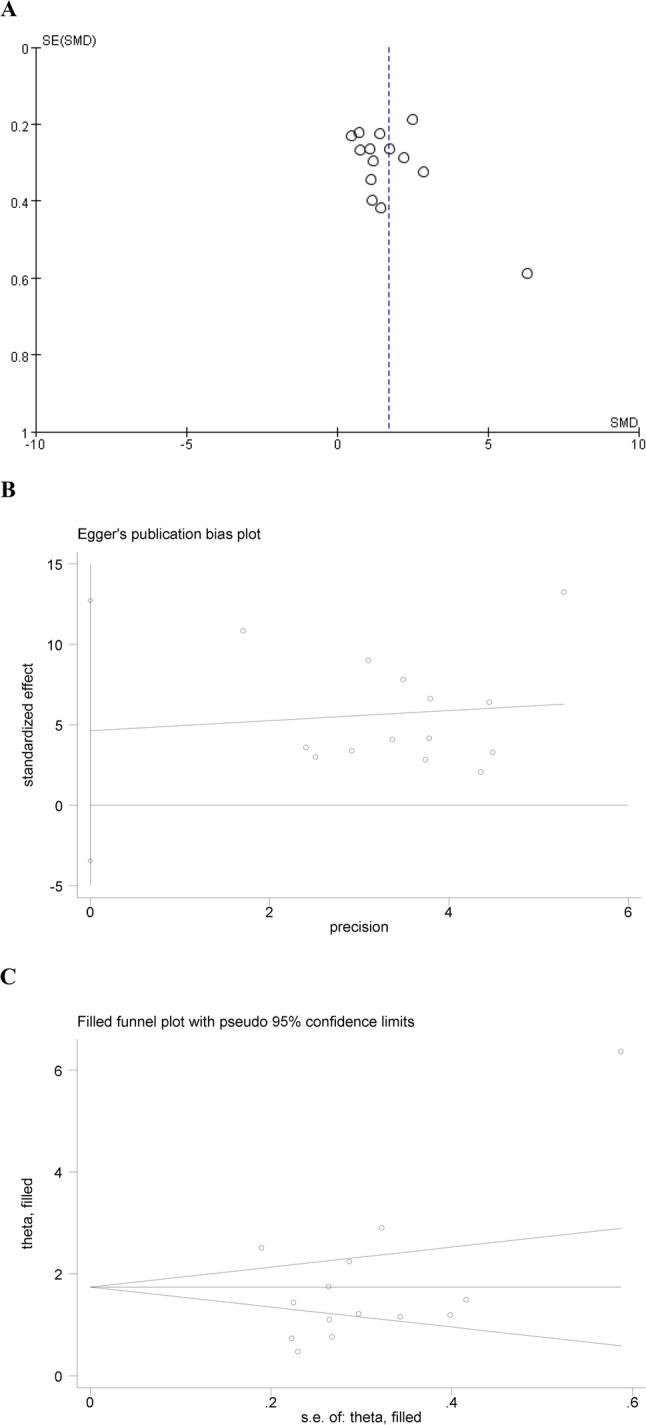
The funnel plot (**A**), Egger’s test (**B**), and trim and fill plot (**C**) of practical skill scores. SE = standard error, SMD = standardized mean difference

Funnel plot, Egger’s test, and the trim-and-fill method were used to evaluate publication bias within the meta-analysis of theoretical knowledge scores and practical skill scores. As indicated in Figs. 8 and 9, funnel plots of both theoretical knowledge scores and practical skill scores demonstrated the asymmetric distribution of scattered dots, but Egger’s tests were not significant (*p* = 0.43 and 0.24, respectively). Furthermore, the trim-and-fill method was employed, which did not impute any missing studies on either side of the funnel plots, indicating that the observed asymmetry did not materially affect the overall effect estimate (Figs. 8 and 9). Consequently, the pooled effect size remained unchanged after adjustment. Taken together, no evidence of significant publication bias existed in the current meta-analysis.

## Discussion

In the current meta-analysis, a comprehensive literature search for comparisons of TBL and LBL teaching methods in Chinese neurology education was conducted, and 16 studies were included. Our results indicated that the students participating in the TBL intervention performed better on theoretical knowledge scores and practical skills scores compared to students who had undergone traditional LBL. Despite the substantial heterogeneity that existed in the analyses, leave-one-out sensitivity analyses revealed that no single study drove the pooled results. Furthermore, visual inspection of the funnel plots revealed asymmetry, but both Egger’s tests and trim-and-fill plots (which yielded no imputed studies) indicated an absence of significant small-study effects, and thus there was no statistical evidence that our results were biased by the non-publication of small, non-significant studies. The overall effect estimate can be considered robust. In addition, secondary outcomes demonstrated significant improvements in self-study ability, communication skills and teamwork ability, highlighting the multifaceted benefits of TBL. Meanwhile, students who received TBL exhibited higher levels of satisfaction with the teaching effects.

Traditional teaching modality in Chinese medical education is the LBL model, which is characterized by teacher-centricity and the passive acquisition of knowledge by learners [32]. This one-way and didactic approach limits student participation and fails to develop students’ creativity and clinical thinking skills [33]. In contrast to LBL, TBL is a student-centered teaching approach [34]. The learning process of TBL includes three stages: preparation, readiness assurance, and application [34]. Generally, students need to prepare the tasks on the designated topic before class. Then, students would begin their session by taking an individual test and a team-based test, followed by immediate feedback. Afterwards, students would complete a series of group tasks designed by the teacher, and present their assignments and share their constructive thoughts [35]. In this educational strategy, a positive connection is built between learners and the educator, which is beneficial to reinforce student engagement and make the teaching process enjoyable, further deepening the understanding of medical knowledge [34, 36]. As we know, modern medical education should emphasize active learning, collaboration, and experiential practice to foster students’ capacity for independent critical thinking and the application of knowledge in clinical settings [6]. Especially, neurology, with its complex neuroanatomy and reliance on deductive reasoning, is often tougher for learners. Furthermore, neurology is deeply interconnected with diverse basic medical disciplines like anatomy, pathology, and diagnostics, which makes teaching neurology more challenging. The traditional LBL teaching model, which primarily involves the one-way transmission of theoretical knowledge, often fails to foster students’ independent thinking or to offer the practical application of theoretical knowledge [37]. In contrast, TBL moves neurology education beyond the passive transmission of knowledge into the active cultivation of clinical neurologists. Students are encouraged to apply knowledge of neuroanatomy and pathophysiology to solve a puzzle in clinical practice. Additionally, modern neurology is a collaborative field, and TBL builds essential interprofessional skills from the beginning, fostering students’ teamwork and communication abilities, which are critical skills for effective collaborative medical care planning. Therefore, TBL contributes to enhancing neurology education by improving medical students’ theoretical knowledge, practical skills as well as core competencies, leading to more well-rounded learning outcomes.

The results of our meta-analysis suggested the TBL method improved both theoretical knowledge scores and practical skill scores in neurology teaching, in line with findings from prior studies across different medical disciplines. Lang et al. [12] conducted a systematic review and meta-analysis to evaluate the teaching effect of the TBL approach on Chinese pharmacy education, and observed that compared to traditional LBL pedagogy, TBL significantly enhanced the theoretical test scores of Chinese pharmacy students. Similar results have been found in the research by Wang et al. [38], which indicated that integrating TBL into standardized dental residency training remarkably enhanced participants’ theoretical scores. Additionally, a recent study on the effects of TBL teaching on nursing education revealed that compared to conventional teaching, TBL teaching enhanced nursing students’ theoretical performance and practical skills [11]. In TBL teaching method, the initial individual test forces students to retrieve information from their memory of scientific materials like neural pathways given in advance, which makes the information more memorable. Furthermore, in TBL, students are provided with real-world clinical problems, like how to diagnose and treat a patient with dizziness, and they must apply their theoretical knowledge to solve these problems, moving beyond memorization to actual use, which not only solidifies their own mastery of the theoretical knowledge but also develops students’ essential practical skills. Altogether, TBL leads to a deeper and more flexible understanding of theoretical knowledge, which directly translates to higher performance of students on exams. Meanwhile, TBL also effectively bridges the gap between theoretical knowledge and practical application, which is the core challenge of clinical training.

The Chi2 test indicated substantial heterogeneity for the meta-analyses of both theoretical knowledge and practical skill scores. High heterogeneity in educational intervention meta-analyses is common, since variations in teaching protocols, outcome measurement tools, student populations as well as course durations among different studies are inevitable. In the present meta-analysis, we employed meta-regression analysis to identify the causes of heterogeneity, which indicated that intervention methods, intervention duration and types of participants failed to account for the observed heterogeneity. Furthermore, the results of the subgroup analyses supported the findings of meta-regression analyses. In the subgroup analyses, the high *I*^2^ values persisted in nearly all strata, suggesting none of the predefined subgroup variables (including intervention methods, intervention duration and types of participants) could fully explain the obvious heterogeneity observed across studies. Hence, our findings should be interpreted with considerable caution, and the true effects of TBL-based approaches may vary across different educational contexts.

Furthermore, subgroup analyses were conducted to assess differences in effects of theoretical knowledge and practical skills based on intervention duration, intervention methods, and types of participants. The subgroup analysis by intervention duration indicated that studies with longer durations (> 4 weeks) yielded significantly larger effect sizes than those with shorter durations(≤ 4 weeks). The findings suggest that the effectiveness of TBL teaching may be time-dependent, with prolonged exposure leading to cumulative benefits. Notably, most of included studies did not report the intervention durations, but this subgroup exhibited the largest pooled effect size. This may be because studies with unreported durations often employed less rigorous designs, which are known to inflate effect sizes, or because these studies implemented longer durations. Thus, curriculum designers could consider allocating sufficient instructional hours to allow the intervention to mature and yield its full potential. In the subgroup stratified by intervention methods (TBL alone vs. TBL combined with other methods), both subgroups indicated that the pooled effect size significantly favored the TBL teaching over traditional lectures, but subgroup differences were not significant, suggesting that combined methods do not necessarily enhance educational effectiveness compared to single method. Several factors may account for this result. First, implementing multiple strategies simultaneously may struggle to execute each with high fidelity, potentially diluting the overall impact, while a well-implemented single method may achieve its intended effect more consistently. Second, the diversity of combined methods and the ambiguity of time proportions devoted to TBL versus other methods may cause considerable heterogeneity within both subgroups, thereby limiting the ability to detect a true difference. Additionally, the subgroup analysis by types of participants indicated that interns or residents achieved slightly greater improvement with the TBL teaching compared to campus-based students. Previous research supports our findings, showing that practical experience allows interns to anchor new information to concrete patient scenarios, which enhances meaningful learning, thus improving learning outcomes [39]. However, this difference failed to reach statistical significance. Given the limited sample size of included studies in our meta-analysis, our results warrant additional studies.

A previous meta-analysis performed by Li et al. [6] compared different teaching methods including TBL in Chinese neurology teaching, which also observed that TBL showed superior performance over traditional LBL in both theoretical and practical skills examinations. However, in our meta-analysis, we included more studies and conducted a more comprehensive analysis based on not only theoretical knowledge and practical skills, but also other abilities. Our meta-analysis revealed that, compared to LBL, TBL remarkably improved students’ self-study ability, communication skills and teamwork ability. The improvement in such abilities may be due to the following reasons. Firstly, in TBL teaching method, the learning materials would firstly be assigned to participants, and they are expected to prepare their assignments on the designated topic before class [34], which could enhance their self-learning ability. Secondly, participants engage collaboratively to complete a series of team tasks, which promotes their communication skills and teamwork ability in this process. Additionally, our meta-analysis found that compared to LBL, TBL obtained higher levels of satisfaction among participants. TBL is an active learning process, and students are encouraged to discuss, debate and apply knowledge, which makes the class time dynamic and intellectually stimulating. We speculate that as students repeatedly practice applying concepts and successfully defend their reasoning, their confidence in their own knowledge and abilities may grow significantly. This self-efficacy is a huge driver of satisfaction.

Of note, TBL has also been implemented in neurology education in countries other than China with promising results. For instance, a study from Germany reported that undergraduates receiving a supplementary TBL-class remarkably improved clinical decision-making skills in neurology, suggesting TBL may be an effective pedagogical approach for fostering clinical reasoning skills in undergraduate neurology education [40]. Additionally, the investigation from Iran indicated that in contrast to conventional lectures, TBL yielded greater academic success and higher levels of student satisfaction among undergraduates in neurology courses [41]. Another research conducted in Singapore demonstrated that TBL was an effective approach for enhancing undergraduate students’ knowledge in neurological localization and neurological emergencies [42]. Even though these studies have shed light on the benefits of TBL for neurology education in diverse students’ populations, meta-analyses and systematic reviews are still needed to provide a higher level of evidence.

Some limitations must be acknowledged in our meta-analysis. Firstly, due to the retrospective nature of the protocol registration on INPLASY, there is a potential risk of reporting bias. Secondly, the studies included in our meta-analysis generally had low methodological quality, with few describing the methods of randomization and none indicating allocation concealment. Consequently, there is a high potential for selection bias, which may lead to an overestimation of the efficacy in educational intervention studies. Future high-quality RCTs with rigorous randomization procedures and allocation concealment are needed to validate our results. Thirdly, considerable heterogeneity across studies may affect the validity of meta-analysis results. Fourthly, in the subgroup analysis based on the intervention methods, our results indicated that TBL combined with other methods enhanced learning outcomes. However, this finding may be ascribed to the alternative intervention rather than TBL, which could be further verified in future studies. Lastly, since all included studies were carried out in mainland China, our findings may be limited by the distinctive cultural, institutional, and pedagogical characteristics of the Chinese educational system. Future investigations based on other educational contexts are needed to determine the generalizability of our results.

## Conclusion

The current meta-analysis suggests that TBL-based teaching approaches (including TBL alone and TBL combined with other methods) in Chinese neurology teaching may be more effective than traditional LBL in enhancing theoretical scores and practical skills. Moreover, TBL may be associated with improved self-study capacity, communication skills, teamwork ability as well as student satisfaction. However, our results should be interpreted with caution due to the mentioned limitations such as the low methodological quality of the included studies and obvious heterogeneity. Future multicenter, cross-cultural and high-quality studies are needed to validate and generalize our findings. 

## Supplementary Information

Supplementary Material 1: PRISMA checklist.

Supplementary Material 2: The search strategy for every database.

Supplementary Material 3: Fig S1. Subgroup analyses of theoretical knowledge scores for TBL compared with LBL.

Supplementary Material 4: Fig S2. Subgroup analyses of practical skill scores for TBL compared with LBL.

## Data Availability

The data can be obtained through contacting the authors.

## Abbreviations

CI: Confidence interval
LBL: Lecture-based learning
TBL: Team-based learning
OR: Odds ratio
SMD: Standardized mean difference
RCTs: Randomized controlled trials

## References

[1] Li X, Li F, Liu W, Xie Q, Yuan B, Wang L, et al. Effectiveness of the application of small private online course combined with PBL model based on massive open online course in the teaching of neurology. BMC Med Educ. 2024;24(1):1518. 39716179 10.1186/s12909-024-06460-5 PMC11667845

[2] Schon F, Hart P, Fernandez C. Is clinical neurology really so difficult? J Neurol Neurosurg Psychiatry. 2002;72(5):557–9. 11971033 10.1136/jnnp.72.5.557 PMC1737866

[3] Murphy S, Carey E, Dablouk L, Alomairi J, Maasarani J, Ong JB, et al. Neurophobia amongst medical students: Hype or reality. Brain Spine. 2024;4:104134. 39687082 10.1016/j.bas.2024.104134 PMC11647157

[4] Jozefowicz RF. Neurophobia: the fear of neurology among medical students. Arch Neurol. 1994;51(4):328–9. 8155008 10.1001/archneur.1994.00540160018003

[5] Matthias AT, Nagasingha P, Ranasinghe P, Gunatilake SB. Neurophobia among medical students and non-specialist doctors in Sri Lanka. BMC Med Educ. 2013;13:164. 24321477 10.1186/1472-6920-13-164 PMC3909313

[6] Li X, Zhang L, Sun W, Lei M, Li Y, Zhang J, et al. Comparison of the effects of different teaching methods on the effectiveness of teaching neurology in China: a bayesian network meta-analysis and systematic review. BMC Med Educ. 2024;24(1):1560. 39736639 10.1186/s12909-024-06397-9 PMC11687189

[7] Lou J, Guo F. Comparing the seminar-case learning and lecture-based learning models in medical education: a meta-analysis of randomized controlled trials. BMC Med Educ. 2025;25(1):470. 40170131 10.1186/s12909-025-07041-w PMC11963456

[8] Thompson BM, Schneider VF, Haidet P, Levine RE, McMahon KK, Perkowski LC, et al. Team-based learning at ten medical schools: two years later. Med Educ. 2007;41(3):250–7. 17316209 10.1111/j.1365-2929.2006.02684.x

[9] Michaelsen LK, Fink LD. Designing effective group activities: lessons for classroom teaching and faculty development. Prof Organizational Dev Netw High Educ. 1997;16:385.

[10] Xie ZB, Cheng XY, Li XY, Zhang YF. Team based learning pedagogy enhances the education quality: a systematic review and meta-analysis. BMC Med Educ. 2025;25(1):580. 40259292 10.1186/s12909-025-07175-x PMC12010525

[11] Xiaoyan W, Lifeng Y, Jing J. Effects of TBL teaching on nursing students’ knowledge, practical skills and core ability: A systematic review and meta-analysis. Nurse Educ Pract. 2024;80:104125. 39317089 10.1016/j.nepr.2024.104125

[12] Lang B, Zhang L, Lin Y, Han L, Zhang C, Liu Y. Team-based learning pedagogy enhances the quality of Chinese pharmacy education: a systematic review and meta-analysis. BMC Med Educ. 2019;19(1):286. 31357986 10.1186/s12909-019-1724-6 PMC6664710

[13] Wu W, Pu L, Zhang E, Xiong S, Zhou X, Xia X, et al. Application of team-based learning to ophthalmology in China. Front Public Health. 2022;10:922325. 36299748 10.3389/fpubh.2022.922325 PMC9589088

[14] Moher D, Liberati A, Tetzlaff J, Altman DG, PRISMA Group. Preferred reporting items for systematic reviews and meta-analyses: the PRISMA statement. BMJ. 2009;339:b2535. 21603045 PMC3090117

[15] Higgins JP, Altman DG, Gøtzsche PC, Jüni P, Moher D, Oxman AD, et al. The Cochrane Collaboration’s tool for assessing risk of bias in randomised trials. BMJ. 2011;343:d5928. 22008217 10.1136/bmj.d5928 PMC3196245

[16] Zhang Y. Application of TBL teaching method in neurology teaching. J Shenyang Med Coll. 2022;24(5):557–60. Chinese.

[17] Lei J, Zhao XF, Ma JH. Control Study on TBL Teaching in Undergraduate Course in the Probation Neurology Teaching. Chin Contin Med Educ. 2016;8(3):23–4. Chinese.

[18] Wang XS, Chen SY, Xu HQ. Using Chinese and Foreign Collaborative TBL teaching Method in preceptorship of Neurology for overseas Students. China High Med Educ. 2017; 9:110–111. Chinese.

[19] Rong W, Xiong J, Zhang J, Wu LZ. Exploration of the application of TBL teaching mode in neurology teaching. EDUCATION MORNIZATION. 2018;1:288. Chinese.

[20] Yin XH. A study on the application of team-based learning strategies in the teaching of intravenous thrombolysis in hyperacute cerebral infarction for physicians in standardised neurology residency training. Cardiovas Dis Electr J Int Trad Chin West Med. 2019;7(18):69. Chinese.

[21] Yang LH, Liu SQ, Xu B, Ye JH, Tao EX. Application of the team-based learning combining lecture-based learning methods in the clinical probation of neurology. BMC Med Educ. 2013;15(3):287–90. Chinese.

[22] Qin H, Qin YY, Jiang Y, Qin FY, Chen DD. The application of Internet + TBL teaching mode in neurology teaching. Zhongguo Keji Jingji Xinwen Shujuku Jiaoyu. 2024;7:194–7. Chinese.

[23] Fan QY, Liu JJ, Wang HQ, Du Y, Ren HW, Gao Z, et al. An investigation of the application of team based learning joint error correction teaching method in the teaching of lumbar puncture surgery. Health Vocat Educ. 2020;38(20):143–5. Chinese.

[24] Zhang X, Wang HP, Zheng YX, Wang ZH, Sun Y. Application of TBL teaching method based on WeChat mini program in internship teaching of pediatric neurology. Western China Qual Educ. 2023;9(20):125–8. Chinese.

[25] Fan QY, Wang HQ, Liu JJ, Du Y, Yao L, Li YL, et al. Application of TBL combined error correction teaching method in the neurological examination teaching. Chin Med Edu Tech. 2020;34(5):661–4. Chinese.

[26] Xu HZ, Cui XP, Ye JX, Chen FS. Study on application of TBL combined with CP teaching method in practical teaching of neurology in undergraduate education. Health Vocat Educ. 2025;43(4):52–5. Chinese.

[27] Gao J, Dai JW. Application of TNS combined with TBL teaching model in undergraduate clinical internship of neurology department. Health Vocat Educ. 2023;41(4):120–3. Chinese.

[28] DF Z, JN D, WH Y. Application of PBL combined with TBL teaching mode in neurology internship. Continuing Med Educ. 2020;34(5):15–7. Chinese.

[29] Wang J, He JS, Chen YB, Cao MY. Application of CBL combined with TBL teaching method in clinical practice of cerebrovascular diseases. J Xiangnan Univ (Med Sci). 2020;22(1):69–71. Chinese.

[30] Liu Y, Li BS, Tang P. Application of flipped classroom combined with TBL in standardized training of neurology resident physicians. J Mod Med Health. 2022;38(21):3743–6. Chinese.

[31] Hou XW, Feng HQ, Wang R. The Application of case-based learning teaching model combined with TBL teaching method in internship teaching of cerebrovascular disease. J Baotou Med Coll. 2021;37(2):125–6. 132 Chinese.

[32] Zhao B, Potter DD. Comparison of Lecture-Based Learning vs Discussion-Based Learning in Undergraduate Medical Students. J Surg Educ. 2016;73(2):250–7. 26572094 10.1016/j.jsurg.2015.09.016

[33] Sakka S. Team-based learning versus traditional lectures in local anesthesia course: A cross-sectional study exploring dental students’ perception and performance. Saudi J Anaesth. 2025;19(3):266–70. 40642624 10.4103/sja.sja_582_24 PMC12240533

[34] Sakka S. Student feedback on team-based learning in a preclinical oral surgery course: A pilot study. J Taibah Univ Med Sci. 2024;19(4):705–10. 39006373 10.1016/j.jtumed.2024.06.002 PMC11246042

[35] Pérez-Guillén S, Carrasco-Uribarren A, Yeung E, Serra-Llobet P, Pardos-Aguilella P, Cabanillas-Barea S. Implementing Team-Based Learning in Physiotherapy Education: Students’ Perceptions and Preferences Compared to the Traditional Lecture. Adv Med Educ Pract. 2025;16:1019–27. 40524723 10.2147/AMEP.S519244 PMC12168947

[36] Burgess A, Haq I, Bleasel J, Roberts C, Garsia R, Randal N, et al. Team-based learning (TBL): a community of practice. BMC Med Educ. 2019;19(1):369. 31615507 10.1186/s12909-019-1795-4 PMC6792232

[37] Zeng HL, Chen DX, Li Q, Wang XY. Effects of seminar teaching method versus lecture-based learning in medical education: A meta-analysis of randomized controlled trials. Med Teach. 2020;42(12):1343–9. 32795244 10.1080/0142159X.2020.1805100

[38] Wang L, Chen P, Wang X, Wei S, Lin J, Jing X. Integrating team-based and peer-teaching strategies for standardized dental residency: a path to active learning and professional growth. BMC Med Educ. 2025;25(1):618. 40287670 10.1186/s12909-025-07023-y PMC12032740

[39] Xiang Y, Liu D, Liu L, Liu IC, Wu L, Fan H. Impact of case-based learning on critical thinking dispositions in Chinese nursing education: a systematic review and meta-analysis. Front Med (Lausanne). 2025;12:1452051. 40166064 10.3389/fmed.2025.1452051 PMC11956162

[40] Jost M, Brüstle P, Giesler M, Rijntjes M, Brich J. Effects of additional team-based learning on students’ clinical reasoning skills: a pilot study. BMC Res Notes. 2017;10(1):282. 28705246 10.1186/s13104-017-2614-9 PMC5512944

[41] Jafari Z. A comparison of conventional lecture and team-based learning methods in terms of student learning and teaching satisfaction. Med J Islam Repub Iran. 2014;28:5. 25250250 PMC4154282

[42] Tan NC, Kandiah N, Chan YH, Umapathi T, Lee SH, Tan K. A controlled study of team-based learning for undergraduate clinical neurology education. BMC Med Educ. 2011;11:91. 22035246 10.1186/1472-6920-11-91 PMC3219570

