# Supplementary material for: TBL alone or combined with other teaching methods for neurology education in China: a meta-analysis of randomized controlled trials
Source: BMC Med Educ. 2026 May 22;26:1154. doi: 10.1186/s12909-026-09468-1 (PMC13371298; doi:10.1186/s12909-026-09468-1)
Supplement: Supplementary file 2 — Supplementary Material 2: The search strategy for every database. [file 12909_2026_9468_MOESM2_ESM.doc]

**The search strategy for every database**

**Pubmed:**

1. team-based learning [Title/Abstract]

2. TBL [Title/Abstract]

3. 1 or 2

4. lecture-based learning [Title/Abstract]

5. LBL [Title/Abstract]

6. traditional teaching [Title/Abstract]

7. 4 or 5 or 6

8. neurology [Title/Abstract]

9. 3 and 7 and 8

**Cochrane Library:**

1. MeSH descriptor: [team-based learning] explode all trees

2. (“team-based learning” OR “TBL” ):ti,ab,kw

3. MeSH descriptor: [lecture-based learning, traditional teaching] explode all trees

4. (“lecture-based learning” OR “LBL” OR “traditional teaching”):ti,ab,kw

5. neurology:ti,ab,kw

6. (1 or 2) and (3 or 4) and 5.

**Web of Science:**

1. TS=(“team-based learning” OR “TBL” )

2. TS=(“lecture-based learning” OR “LBL” OR “traditional teaching”)

3. TS=neurology

4. 1 and 2 and 3

**China National Knowledge Infrastructure (CNKI):**

主题 (基于团队的学习 or 团队学习) AND

主题 (讲授式学习 or 以授课为基础的学习 or 传统教学) AND

主题 (神经病学 or 神经内科)

**Chinese VIP database:**

题名或关键词 (基于团队的学习 or 团队学习) AND

题名或关键词 (讲授式学习 or 以授课为基础的学习 or 传统教学) AND

题名或关键词 (神经病学 or 神经内科)

**Chinese Wanfang Database:**

主题 (基于团队的学习 or 团队学习) AND

主题 (讲授式学习 or 以授课为基础的学习 or 传统教学) AND

主题 (神经病学 or 神经内科)
