# Supplementary material for: TBL alone or combined with other teaching methods for neurology education in China: a meta-analysis of randomized controlled trials
Source: BMC Med Educ. 2026 May 22;26:1154. doi: 10.1186/s12909-026-09468-1 (PMC13371298; doi:10.1186/s12909-026-09468-1)

**Supplementary Figure 2 Subgroup analyses of practical skill scores for TBL compared with LBL. (A) Forest plots of subgroup analysis results based on intervention methods; (B) Forest plots of subgroup analysis results based on duration of intervention; (C) Forest plots of subgroup analysis results based on participants. CI = confidence interval, SD = standard deviation, LBL = lecture-based learning, TBL = team-based learning.**

**A Intervention methods:**


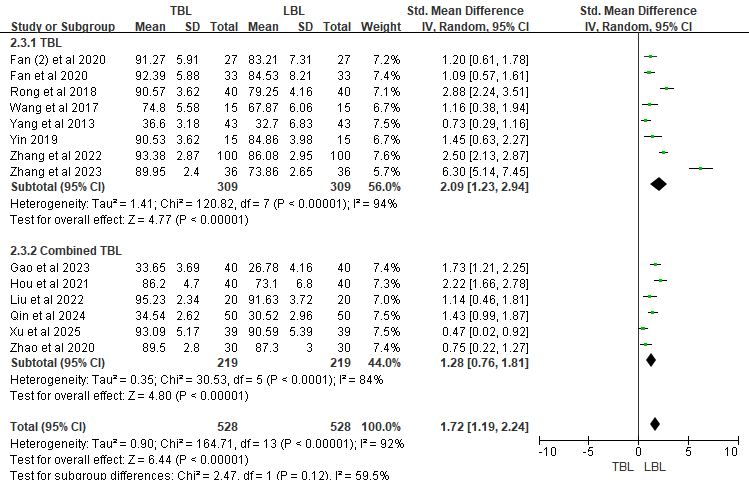


**B Intervention duration:**


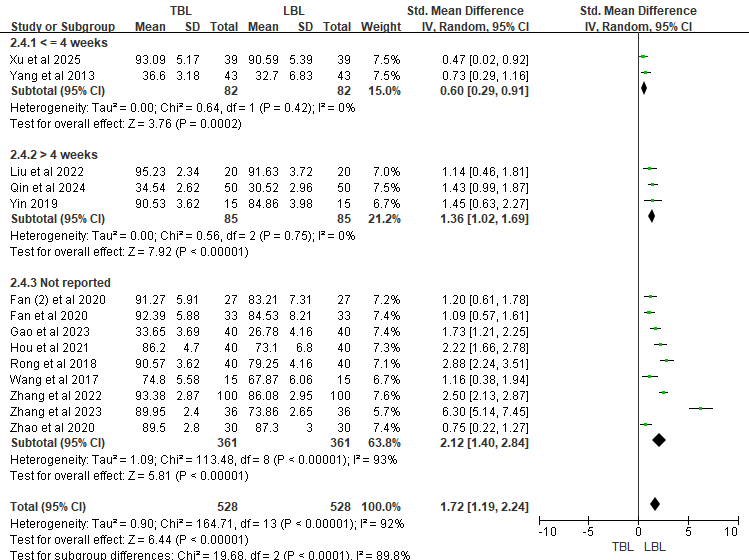


**C Participants:**


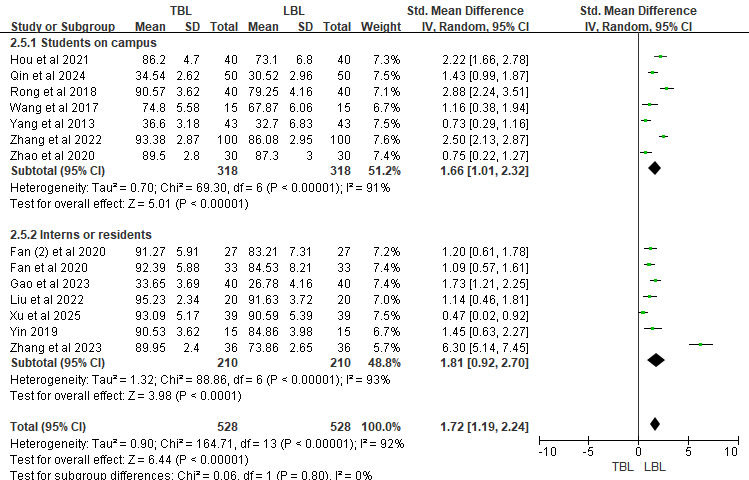

Supplement: Supplementary file 4 — Supplementary Material 4: Fig S2. Subgroup analyses of practical skill scores for TBL compared with LBL. [file 12909_2026_9468_MOESM4_ESM.doc]
